# New distance measure for comparing protein using cellular automata image

**DOI:** 10.1371/journal.pone.0287880

**Published:** 2023-10-05

**Authors:** Luryane F. Souza, Hernane B. de B. Pereira, Tarcisio M. da Rocha Filho, Bruna A. S. Machado, Marcelo A. Moret

**Affiliations:** 1 Centro de Ciências Exatas e das Tecnologias, Universidade Federal do Oeste da Bahia, Barreiras, Bahia, Brazil; 2 Programa de Modelagem Computacional e Tecnologia Industrial, SENAI-CIMATEC, Salvador, Bahia, Brazil; 3 DEDC, UNEB, Salvador, Bahia, Brazil; 4 Instituto de Física, Universidade de Brasília, Brasília, Distrito Federal, Brazil; 5 DCET, UNEB, Salvador, Bahia, Brazil; CIFRI: Central Inland Fisheries Research Institute, INDIA

## Abstract

One of the first steps in protein sequence analysis is comparing sequences to look for similarities. We propose an information theoretical distance to compare cellular automata representing protein sequences, and determine similarities. Our approach relies in a stationary Hamming distance for the evolution of the automata according to a properly chosen rule, and to build a pairwise similarity matrix and determine common ancestors among different species in a simpler and less computationally demanding computer codes when compared to other methods.

## Introduction

Bioinformatics has been growing recently and consolidating itself as a research area, bringing together researchers from different areas such as molecular biology, physics, mathematics, computer science, deep learning technology, and science, among others, to analyze, interpret, and process biological data. Its origins date back to the first protein sequencing studies in the 1950s when insulin was sequenced well before the first microcomputers [1–3]. Databases such as UniProt [4], GenBank [5] provide large amounts of data on DNA, RNA, and protein sequences. At the beginning of 2023, the GenBank [5] website has registered over 240 million sequences. The current main challenge is to analyze this large amount of sequence data and extract information about these genetic sequences’ functions, structures, locations, and evolutionary relationships. More specifically, for the study of proteins, many computational methods are allied in protein sequence analysis [6–11]. A protein is a macromolecule made up of 20 types of amino acids that can represented in its primary form as a string of characters for each amino acid. In the character string representation, important characteristics of the proteins are hidden, so some methods of graphical representation of this sequence can facilitate the analysis of complex behaviors of this sequence. Different approaches for graphical representations of protein sequences were proposed in Refs. [6, 7, 10–18]. The approach we use in the present work is based on cellular automata (CA) images generated using the sequence of a given protein as initial state. Amino acids are encoded into valid entries of a cellular automaton (see for instance Xiao et al. [7]), using a digital code based on the rules of similarity, complementarity, molecular recognition theory and information theory [6]. A coding based on hydrophobicity indices of amino acids was used in a reduced form by Kavianpour and Vasighi [10] to encode protein sequences to extract features from the images and determine the structural class of the protein. Chaudhuri et al. [18] used a coding with an eight-digit binary code based on the analysis of the molecular structure of each amino acid.

Similarities among sequences, i. e. homologous sequences, hold important information, such as similar functions or as an indication of a recent common ancestor [19]. Indeed, evolutionary relationships between protein sequences can be determined from sequence comparison methods [20–25]. Rahman et al. [11] proposed a method for decomposing CA images using wavelet decomposition and used the horizontal image of this decomposition for protein comparison from an image quality metric. The Hamming distance have been successfully used to evaluate the stability of the concentration of soot during controlled combustion of acetylene and natural gas, within the spatiotemporal standards generated by the evolution of the CA-based system [26]. In previous work by some of the authors [25] we used cellular automata imaging of the Spike proteins to compare variants of the SARS-CoV-2 virus using the stationary Hamming distance, and determined the variants that shared recent common ancestors by looking only at the evolution of the distance between the variant CA and the one for the reference protein initially found in Wuhan-China. As a continuation, we propose a method to build the distance matrix between species pairs using the stationary Hamming distance measuring the dissimilarity between different species. We apply this method for different proteins: ND5, ND6, transferrin, and beta-globin. The proposed approach is effective for grouping similar species and, using cophenetic correlation coefficients, building dendrograms similar to those obtained using the p-distance from the package MEGA [27].

Considering that the p-distance measures the differences between two sequences and that information loss may occur when transforming a protein sequence into a cellular automaton, our results confirm that this loss is minimal, and that our methodology can be used in the analysis of similar proteins. Another advantage is that we use a simple comparison metric not requiring more elaborate processing methods or image textures.

## Materials and methods

A Cellular automaton (CA) is a discrete dynamical system in both space and time evolving under a given spatially local rule. Despite their simplicity they often model complex systems [28]. It is defined from five components: *L*, *S*, *N*, *f* and *B*, with *L* a n-dimensional spatial lattice of cells with values $c_{i}^{t}$, *i* = 1, 2, 3, …, *M* at time *t*. Each cell assumes values in the set of possible states *S*. The neighborhood *N* of a given cell *i* is the set of cells considered in the transition rule *f*. Finally, the boundary conditions of the automaton is represented by *B*.

**Fig 1:**
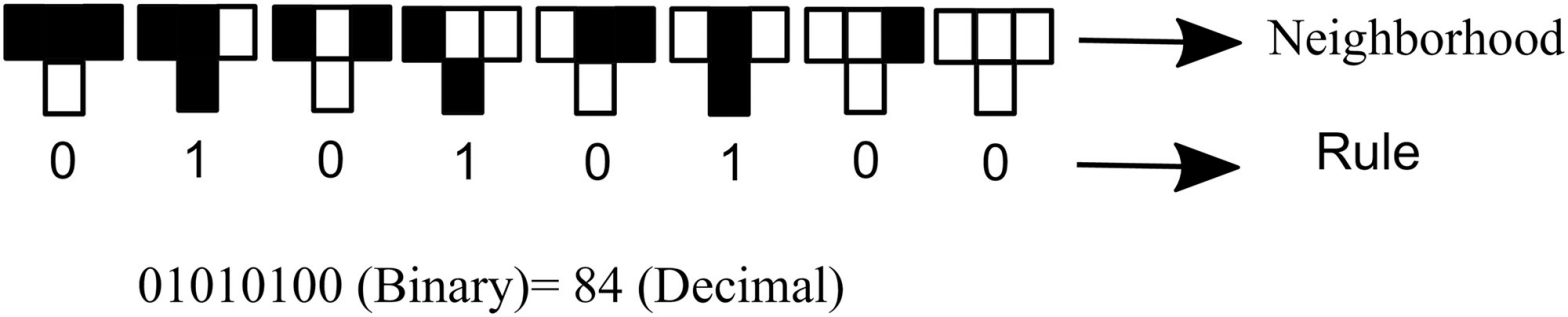
Cellular automaton rule *n*^*o*^ 84 with for the eight types of neighborhoods in the cellular automaton. Rule 84 generated all the cellular atutomata images in this paper.

**Fig 2:**
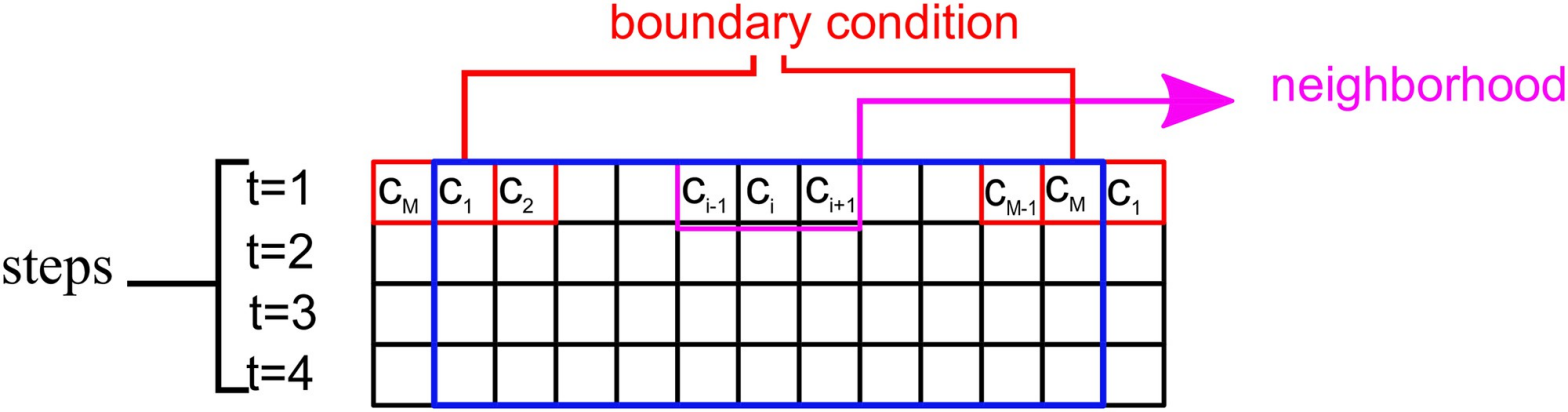
Cellular automaton image.

Here we consider in the presen work one-dimensional cellular automata with a neighborhood of cell *i* it given by the cells *i* − 1, *i* and *i* + 1:

$$N\left(c_{i}^{t}\right)=\left\{c_{i-1}^{t},c_{i}^{t},c_{i+1}^{t}\right\}\;\;i=1,2,\ldots ,M.$$

and a set of two possible states *S* = {0, 1}. Therefore, we have a total of 2^3^ = 8 different possible neighborhoods. The transition function *f* expresses the state assumed by each cell in the next time step according to its neighborhood as a query list for each possible neighborhood state. We thus have a total of 2^8^ = 256 possible evolution rules for the cellular automaton, each rule enumerated 0 to 255 given by the decimal form of it binary representation, as exemplified in Fig 1. The rule used for the evolution of cellular automata throughout this paper was rule 84 (represented in Fig 1), this rule is the best to describe proteins [7]. The boundary condition *B* determining the neighborhood of cells at the extremities of the cellular automaton can be of four types: fixed contour, random, periodic and reflecting [29]. We consider here periodic boundary conditions such that that *c*_*M*+1_ = *c*_1_ and *c*_0_ = *c*_*M*_. Fig 2 illustrates the procedure of forming an image of the cellular automaton composed by the lines for each discrete time value *t* according to the evolution rule.

Since we use a two state cellular automaton, each amino acid will be encoded in a binary code, with different possibilities studied in the literature [6, 10, 18, 30]. For the present study we use the code proposed by Chaudhuri et al. [18] and shown in Table 1, which encodes each amino acid with an 8-digit code based on the molecular structures of amino acids. The coding in Table 1 considers the molecular structure of the amino acid side chains and their non-H (hydrogen) atoms. The 8-digit codes are built based on the amount of C (carbon), N (nitrogen), O (oxygen), and S (sulfur) atoms in the side chain, the presence of an aromatic ring, and covalent bonds between atoms, for more details on the construction of this code see by Chaudhuri et al. [18]. Among other possibilities, this choice is justified by the fact that it yields a better grouping of close species.

**Table 1:** Encoding of amino acids, deletions and missing protein sequence data after alignment. Code based on molecular structure of amino acid side chains by Chaudhuri et al. [18].

Aminoacid	Code	Aminoacid	Code
Glycine (G)	00000000	Cysteine (C)	01000100
Alanine (A)	00000100	Threonine (T)	00110100
Proline (P)	00100110	Asparagine (N)	00101110
Valine (V)	00010110	Glutamine (Q)	00101111
Methionine (M)	00110110	Tyrosine (Y)	10100100
Tryptophan (W)	10110110	Histidine (H)	01111110
Phenylalanine (F)	10000100	Lysine (K)	00110111
Isoleucine (I)	00011110	Arginine (R)	01111111
Leucine (L)	00010111	Aspartic Acid (D)	01110100
Serine (S)	00100100	Glutamic Acid (E)	01110110
Deletion (-)	11111111	Missing information (?)	11111110

**Fig 3:**
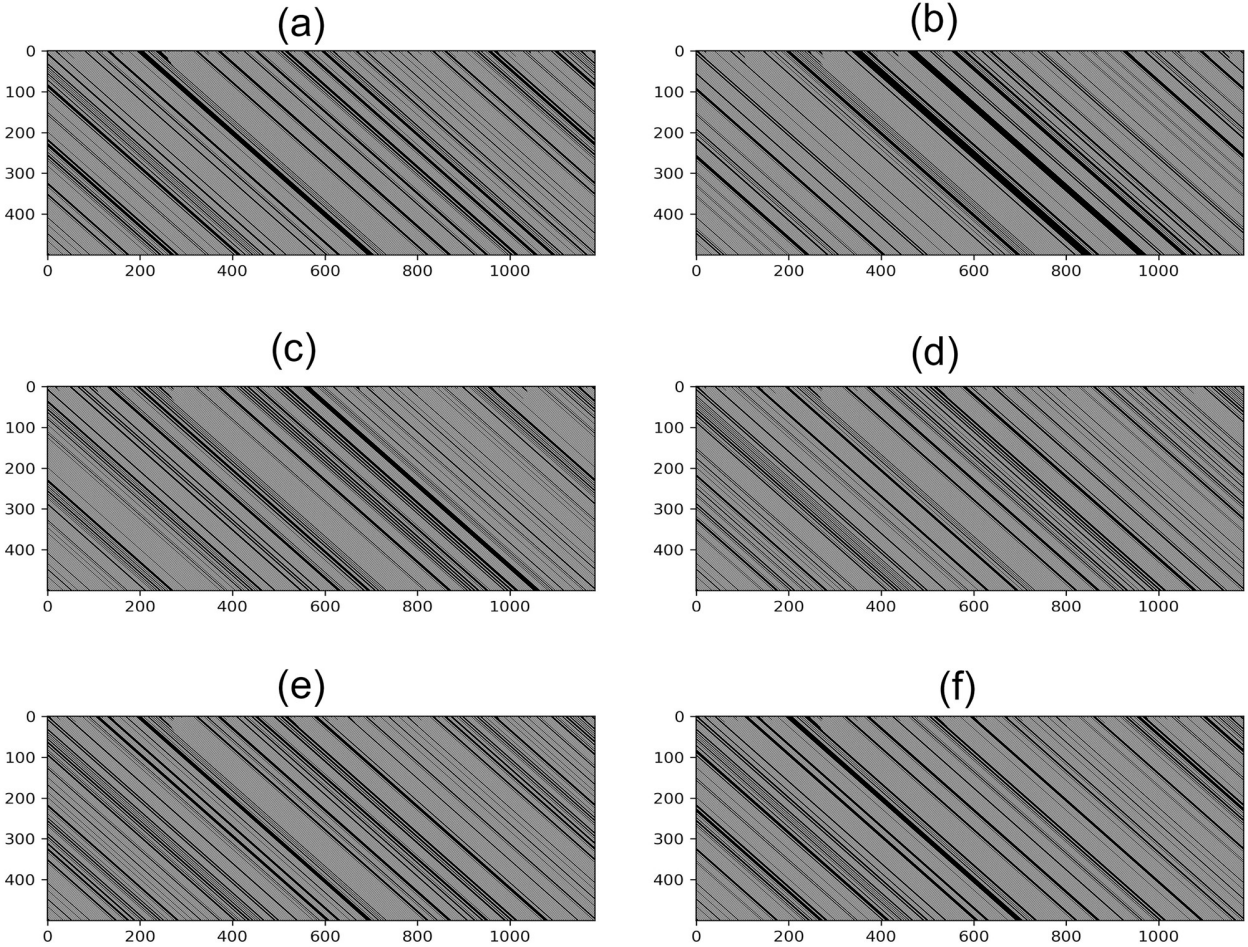
Cellular automaton image of the Beta-Globin protein for A) Human, B) Shark, C) Catfish, D) Turtle, E) Swift and F) Coyote species.

The first step is to align the different protein sequences considered such that each associated automata are of the same size. Deletions and losses are identified and properly represented in each automaton by the respective codes in Table 1. Considering a protein of size *P*, the initial condition will have a size of *M* = 8 × *P*. As a first illustration of our approach we show in Fig 3 the cellular automaton image of Beta-Globin protein for six different animal species, for a total evolution of *t* = 500 steps (this value will be used for the remaining of the present paper). The temporal evolution of the cellular automaton transforms the sequence of characters that hides information from this protein into an image that carries many important characteristics of this protein. For this reason, the image of the cellular automaton is used in several works [6, 7] to model the complex behavior of proteins.

The images of the associated cellular automata provide signatures for different proteins and are used to determine similarities/differences between species. A comparison between those images can be performed with a low computational cost using the Hamming distance from information theory [31] given by the number of changes needed to transform one sequence into another, and implement for the generated images of two cellular automata *CA*_*A*_ and *CA*_*B*_ as:

$$\begin{array}{ccc} D_{H}\left(t\right) & = & \frac{1}{M}\sum_{i=1}^{M}\left|a_{i}^{t}-b_{i}^{t}\right|, \end{array}$$ (1)

with $a_{i}^{t}$ and $b_{i}^{t}$ the values of the *i*th cells of automata *CA*_*A*_ and *CA*_*B*_ at step *t*, respectively. The size of the automaton is *M* = 8 × *P*, where *P* is the size of the aligned protein sequence. As shown in the next section the Hamming distance saturates after a relatively small number of steps. We denominate this saturated value the Stationary Hamming Distance (SHD), which is then used to build the similarity matrix.

## Results and discussion

We apply our approach to the following four protein sequences: beta-globin, NADH Dehydrogenase 5 (ND 5), NADH Dehydrogenase 6 (ND 6) and transferrin. This choice was motivated by the requirement to be able to perform comparisons with previous results in the literature [13, 32]. All sequences were aligned using the ClustalW system [27, 33]. Our results are then compared to those obtained from pairwise p-distance from ClustalW.

### NADH Dehydrogenase 5 (ND 5)

**Fig 4:**
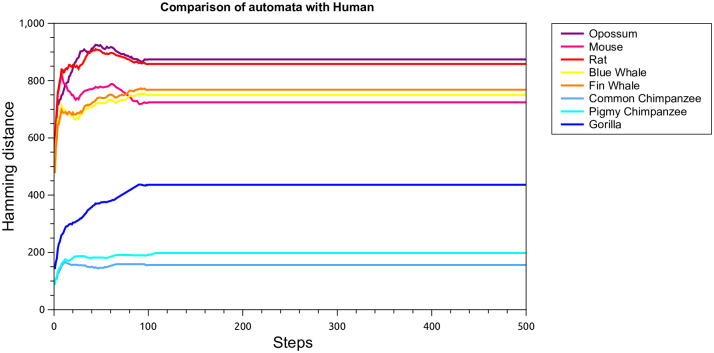
Hamming distance between the cellular automata image for some different mammalian species and Human, as a function of the number of steps.

Protein ND 5 is a sub-unit of the mitochondrial respiratory enzyme complex I (NADH: ubiquinone oxidoreductase) [34], and is responsible for mitochondrial electron transport. Mutations and defects in this enzyme can cause Leigh’s disease and MELAS syndrome. Being highly conserved in eukaryotes, we use data from these sequences to analyze similarities between mammalian species. We consider here the following nine species: Human, Gorilla, Pigmy Chimpanzee, Common Chimpanzee, Fin Whale, Blue Whale, Rat, Mouse, and Opossum. All sequences were taken from the NCBI protein database [5], and their identifications are given in S1 Table in S1 File. The aligned sequences have 613 entries each, and are represented using the coding in Table 1, resulting into a binary sequence of length 4904 for the initial condition of the cellular automaton. The cellular automata image is the generated from the prescription in the previous section and the Hamming distance in Eq (1) between two species as a function of the number of steps. The results for the distance between each of the nine species and Humans are shown in Fig 4.

**Fig 5:**
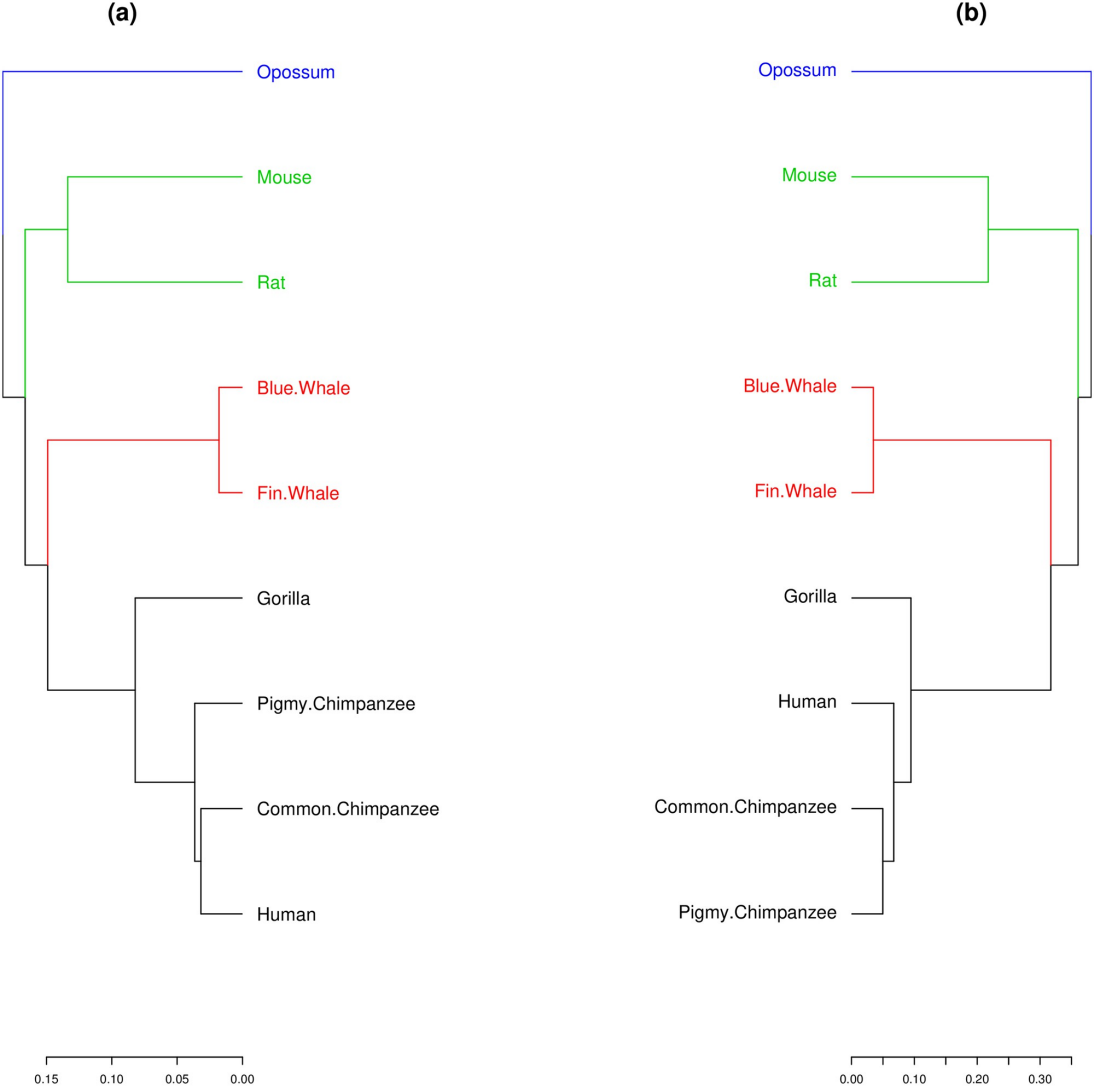
Dendrograms from the ND 5 protein from (a) SHD and (b) p-distance. The four families are well grouped in both dendrograms: Didelphidae (blue), Muridae (green), Balaenopteridae (red), and Hominidae (black).

We then build distance matrices from the SHD and the p-distance, and obtain corresponding dendrograms using the average method from R studio hierarchical grouping [35, 36], and shown in Fig 5. Both dendrograms are identical, with the exception of a small difference of the closest relative of human. Nevertheless both methods correctly group families: Hominidae, Balaenopteridae, Muridae, and Didelphidae. Other methods such as [32] yield dendrograms identical to the one obtained from our method. The cophenetic correlation coefficient [37] between the two dendrogram in Fig 5 is 0.9940, indicating a very close similarity.

### NADH Dehydrogenase 6 (ND 6)

**Fig 6:**
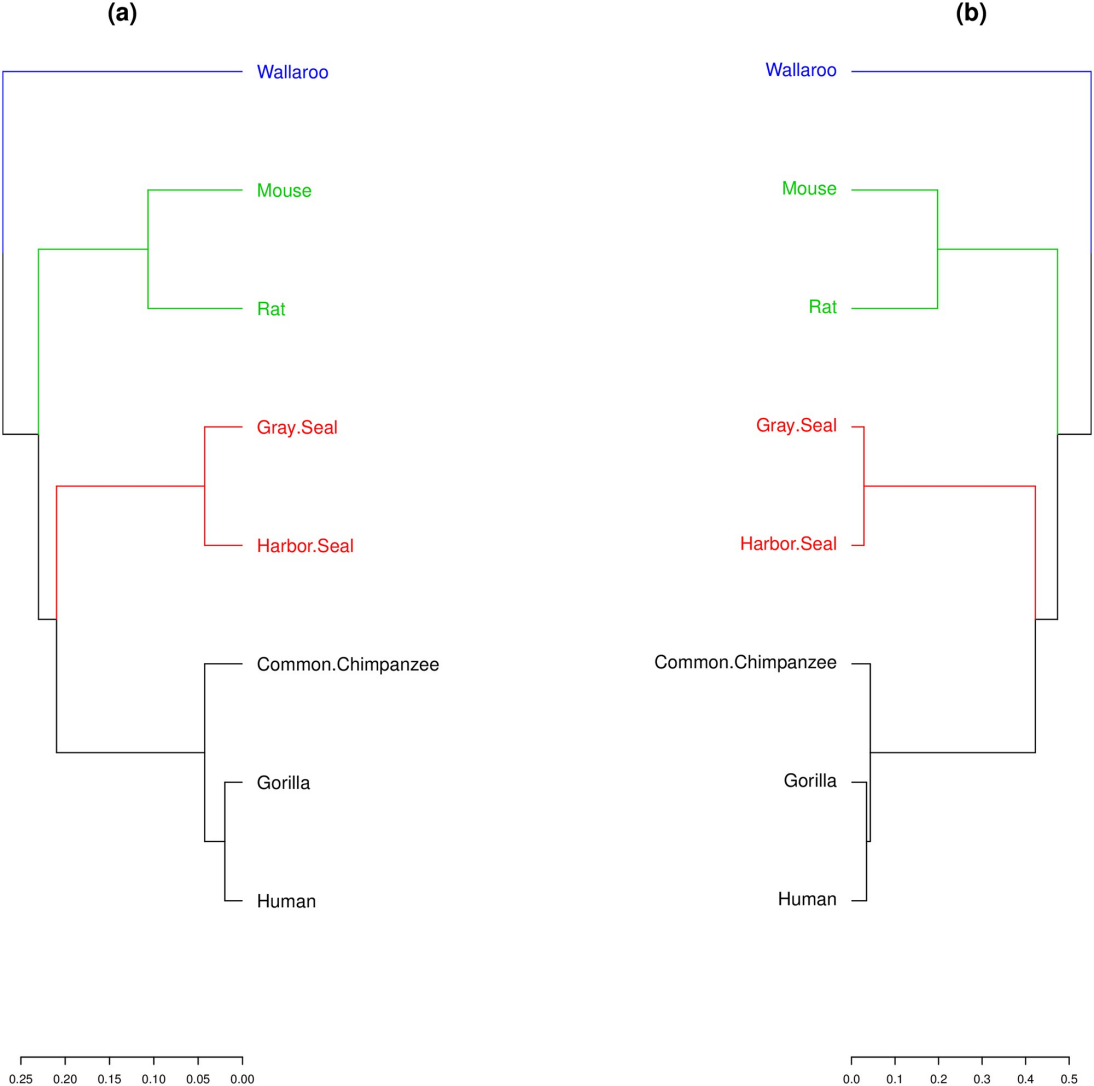
Dendrograms from the ND 6 protein obtained from distance matrices using (a) SHD and (b) p-distance, with the indication of family groupings: Macropodidae (blue), Muridae (green), Phocidae (red), and Hominidae (black).

The NADH Dehydrogenase 6 (ND 6) protein is a sub-unit of the NADH dehydrogenase (ubiquinone) enzyme, located in the mitochondrial inner membrane. Mutations or errors in their sequences can cause Leigh’s disease and spinal muscular atrophy [38]. Protein sequences were obtained from NCBI [5], and their identifications are given in S2 Table in S1 File. The aligned sequences have 176 positions, and thence the initial condition for this protein has 8 × 176 = 1408 cells. We follow the same procedure as for the previous case: the SHD between each pair among the following species: Human, Gorilla, Common Chimpanzee, Gray Seal, Harbor Seal, Rat, Mouse, and Wallaroo. Dendrograms are then obtained from the distance matrices using the SHD and the p-distance, and shown in Fig 6. The two dendrograms are identical and both methods group families correctly: Macropodidae, Muridae, Phocidae, and Hominidae. We note that other methods as alignment-free similarity analysis [32] cannot separate the Macropodidae family from the Muridae. The cophenetic correlation coefficient between the two dendrograms in Fig 6 is 0.9797, indicating again a very close similarity.

### Transferrin

**Fig 7:**
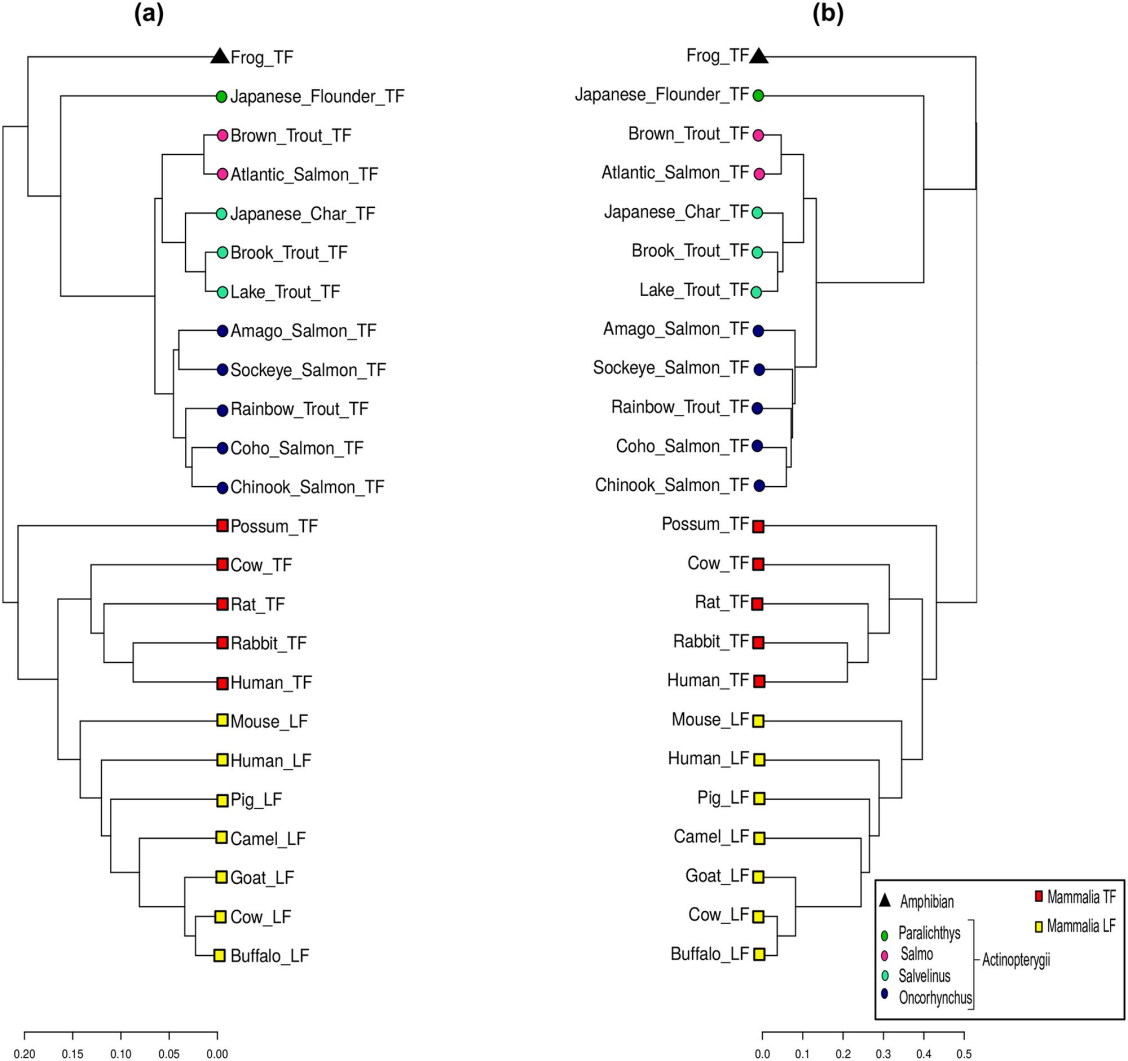
Transferrin protein dendrograms from (a) SHD and (b) p-distance distance matrices.

Transferrin is an iron-binding protein keeping iron at a low concentration in biological fluids. Serum transferrin (TF) is present in mammals, amphibians, and fish [39], and plays an essential role in fighting bacterial infections in fish [40]. Blood iron overload is a rare condition that characterizes hereditary atransferrinemia [41]. We consider a set of 24 transferrin protein sequences across Mammalia, Amphibian, and Actinopterygii species from the NCBI database [5] and their identifications are given in S3 Table in S1 File. The aligned sequences have 750 positions. Each one is then encoded into a binary code of size 8 × 750 = 6000. The dendrogram obtained from the SHD and p-distance are shown in Fig 7. Our approach correctly classifies all species into their respective groups: Mammalia, Amphibian and Actinopterygii and separately grouping mammals’ serum transferrin (TF) and lactotransferrin (LF). It also group correctly species from the genus Salmo (Brown Trout, Atlantic Salmon), Salvelinus (Japanese Char, Brook Trout, Lake Trout), and Oncorhynchus (Amago Salmon, Sockeye Salmon, Rainbow Trout, Coho Salmon, Chinook Salmon) in the Actinopterygii class. Only Amago Salmon (TF) and Sockeye Salmon (TF) were grouped differently, but the same problem has already been reported in previous works [40]. The cophenetic correlation coefficient between the two dendrograms is 0.9671, again indicating a very good similarity between the clusters.

### Beta-Globin

**Fig 8:**
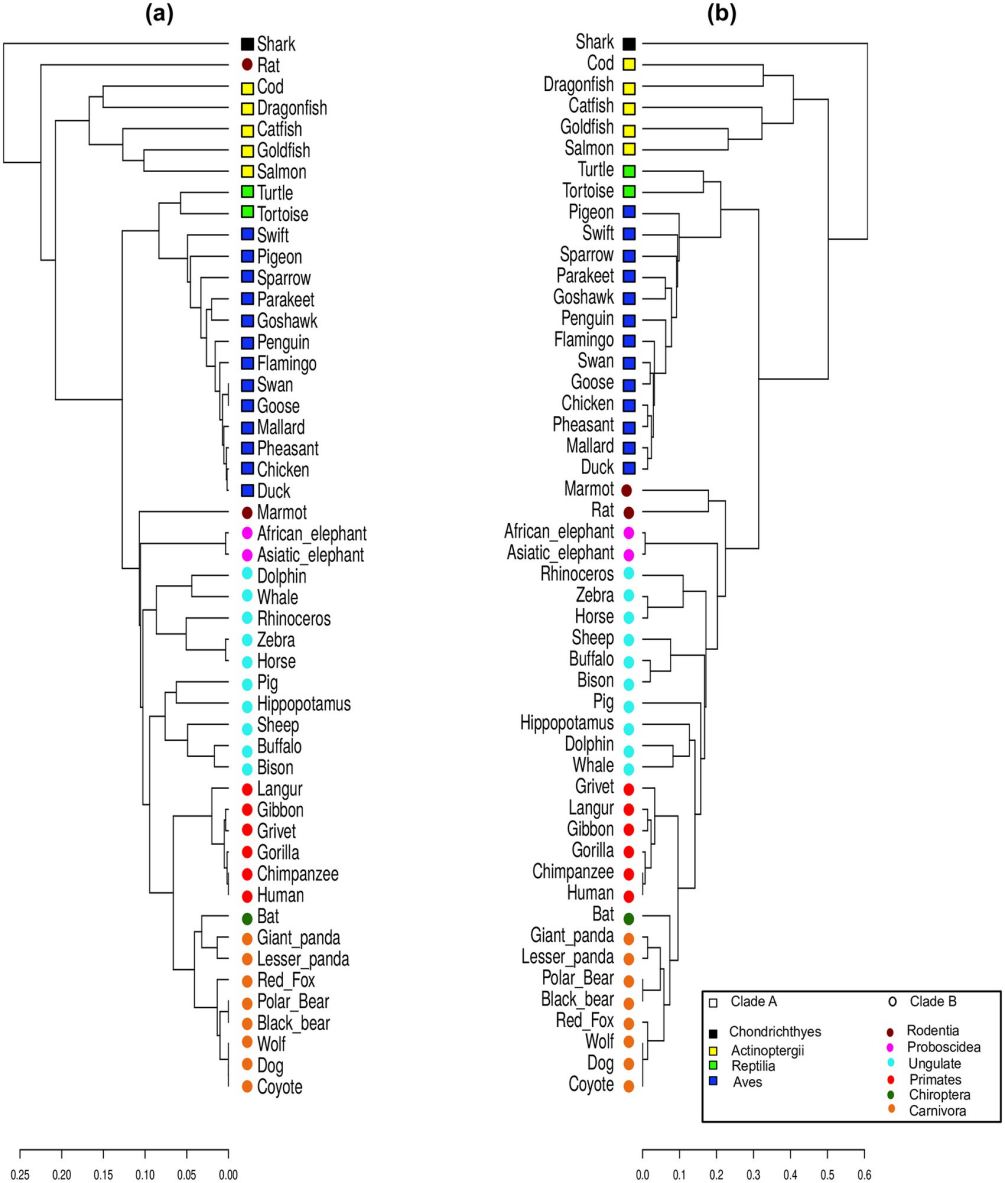
Beta-globin protein dendrograms from SHD (a) and p-distance (b) values. Clade A is represented by squares and Clade B by circles. Different animal groups are represented by colors.

Hemoglobin comprises two chain pairs *α* and *β*, which have distinct chains of amino acids, two dimers of *α* − *β* form hemoglobin. Its principal function is to carry oxygen from blood to tissues. Mutations in the beta-globin chain can cause sickle cell anemia [42]. We consider here 50 beta-globin sequences from different species taken from the NCBI database [5], and their identifications are given in S4 Table in S1 File. The aligned sequences have 148 positions. The initial condition is then coded in 8 × 148 = 1184 cell digits. The corresponding distance matrices for SHD and p-distance then have 50 × 50 = 2500 entries, and the corresponding dendrograms are shown in Fig 8.

Our approach yields a consistent classification of the identified clusters. At variance the results in [13] and the dendrogram obtained using the p-distance, that separates mammals from non-mammals, our approach failed in this point, with rat classified in Clade A. This same inconsistency was also observed in a previous work [32]. Other divergences of the method involve some more recent families but keep the tree similar to the one obtained using the p-distance. These discrepancies observed for Beta-Globin may be due to the fact that we considered a significant and more diverse number of species, and is reflected in the value of 0.8790 for the cophenetic correlation coefficient between the two dendrograms, clearly below the other proteins considered here, but still acceptable.

## Conclusion

We discussed and showed that Cellular automata are a tool for visual comparing of protein sequences and for determining their similarity. We expanded the use of this tool by introducing the use of the Hamming distance from information theory, in order to compare the cellular automata images obtained. Our approach allows to determine phylogenetic relations among species with a good accuracy if one considers that one protein was used in each of the dendrogram presented, but nevertheless has some limitations. We applied it to lysozyme protein sequences (not shown here), with inconclusive results, with the possible explanation that the sequences for this cases are not homologous but are the result of convergent evolution. In this case, the resulting dendrograms from both our method and by using p-distance cannot approximate species with recent common ancestors.

The method’s main advantage is that the cellular automaton is a graphical method; thus, many complex sequence behaviors can be analyzed using the image of the cellular automaton associated with the protein. Furthermore, the Hamming distance is an intuitive metric for measuring the distance between pairs of protein cellular automata, so our method can be the first approach professionals use to analyze fast information from sets of protein sequences. The main disadvantage of the method is that it is not free of alignment. The sequences must be aligned so that the automata have the same size to calculate the Hamming distance. But compared to methods that use alignment, such as p-distance, which also builds the distance matrix, the main advantage of our method is that it codes each amino acid according to its structure so that similar amino acids have closer codes. Thus, our distance measure will give different weights to different mutations. As also observed in [25], who used a code based on the hydrophobicity of amino acids, the sequences that underwent modifications with a change in hydrophobicity had a greater distance and a different weight for each type of mutation.

The complexity of this method can be calculated from the complexity of the three steps of the method. The first step is the generation of cellular automata, which has complexity *O*(*N*^2^), the step of calculating the distances between the automata has complexity *O*(*N*^2^), and the last step, which is the construction of the dendrograms in their groupings we use the UPGMA method to group the species and this method has complexity *O*(*N*^2^). Considering the computational costs of the three steps, the proposed method has complexity *O*(*N*^2^). Thus, the proposed method has the lowest possible cost.

Other approaches use textures from images [11] to compare cellular automata. Ours requires a low computational cost and no processing methods or image textures, with an efficient protein comparison. As a first work we used an evolution rule previously proposed in the literature, but in forthcoming research we will consider other possibilities, and are currently investigating the possibility of coding proteins using the hydrophobicity scale proposed by Moret and Zebende [43].

## Supporting information

S1 File (PDF)

## References

[1] Sanger F, Thompson EOP. The amino-acid sequence in the glycyl chain of insulin. 1. The identification of lower peptides from partial hydrolysates. Biochemical Journal. 1953;53(3):353–366. doi: 10.1042/bj0530353 13032078 PMC1198157

[2] Sanger F, Thompson EOP. The amino-acid sequence in the glycyl chain of insulin. II. The investigation of peptides from enzymic hydrolysates. The Biochemical journal. 1953;53(3):366–374. doi: 10.1042/bj0530366 13032079 PMC1198158

[3] Gauthier J, Vincent AT, Charette SJ, Derome N. A brief history of bioinformatics. Briefings in Bioinformatics. 2018;20(6):1981–1996. doi: 10.1093/bib/bby063 30084940

[4] UniProt. The Universal Protein Resource; 2021. Available from: https://www.uniprot.org.

[5] GenBank. National Center for Biotechnology Information; 2021. Available from: https://www.ncbi.nlm.nih.gov/genbank.

[6] Xiao X, Shao S, Ding Y, Chen X. Digital coding for amino acid based on cellular automata. In: 004 IEEE International Conference on Systems, Man and Cybernetics (IEEE Cat. No.04CH37583). vol. 5; 2004. p. 4593–4598.

[7] Xiao X, Shao S, Ding Y, Huang Z, Chen X, Chou KC. Using cellular automata to generate image representation for biological sequences. Amino Acids. 2005;28:29–35. doi: 10.1007/s00726-004-0154-9 15700108 PMC7088382

[8] Moret MA, Santana MC, Nogueira E, Zebende GF. Protein chain packing and percolation threshold. Physica A. 2006;361:250–254. doi: 10.1016/j.physa.2005.08.001

[9] Moret MA. Self-organized critical model for protein folding. Physica A. 2011;390:3055–3059. doi: 10.1016/j.physa.2011.04.008

[10] Kavianpour H, Vasighi M. Structural classification of proteins using texture descriptors extracted from the cellular automata image. Amino Acids. 2017;49:261–271. doi: 10.1007/s00726-016-2354-5 27778167

[11] Rahman MM, Biswas BA, Bhuiyan MIH. Protein Similarity Analysis by Wavelet Decomposition of Cellular Automata Images. In: 2019 International Conference on Electrical, Computer and Communication Engineering (ECCE); 2019. p. 1–6.

[12] Mu Z, Wu J, Zhang Y. A novel method for similarity/dissimilarity analysis of protein sequences. Physica A: Statistical Mechanics and its Applications. 2013;392(24):6361–6366. doi: 10.1016/j.physa.2013.08.008

[13] Mu Z, Yu T, Liu X, Zheng H, Wei L, Liu J. FEGS: a novel feature extraction model for protein sequences and its applications. BMC Bioinformatics. 2021;22(297). doi: 10.1186/s12859-021-04223-3 34078264 PMC8172329

[14] Liao B, Liao B, Sun X, Zeng Q. A Novel method for similarity analysis and protein sub-cellular localization prediction. Bioinformatics. 2010;26(21):2678–2683. doi: 10.1093/bioinformatics/btq521 20826879

[15] Wu ZC, Xiao X, C CK. 2D-MH: A web-server for generating graphic representation of protein sequences based on the physicochemical properties of their constituent amino acids. J Theor Biol. 2010;267(1):29–34. doi: 10.1016/j.jtbi.2010.08.007 20696175

[16] Xiao X, Wang P, Chou KC. Cellular automata and its applications in protein bioinformatics. Curr Protein Pept Sci. 2011;12(6):508–519. doi: 10.2174/138920311796957720 21787298

[17] Wang M, Yao JS, Huang ZD, Xu ZJ, Liu GP, Zhao HY, et al. A new nucleotide-composition based fingerprint of SARS-CoV with visualization analysis. Medicinal Chemistry. 2005; p. 39–47. doi: 10.2174/1573406053402505 16789884

[18] Chaudhuri PP, Ghosh S, Dutta A, Choudhury SP. Cellular Automata (CA) Model for Protein. Singapore: Springer Singapore; 2018. Available from: 10.1007/978-981-13-1639-5_5.

[19] Pearson WR. An Introduction to Sequence Similarity (“Homology”) Searching. Current Protocols in Bioinformatics. 2013;42(1):3.1.1–3.1.8. doi: 10.1002/0471250953.bi0301s42 23749753 PMC3820096

[20] Lipman DJ, Pearson WR. Rapid and Sensitive Protein Similarity Searches. Science. 1985;227(4693):1435–1441. doi: 10.1126/science.2983426 2983426

[21] Campanella JJ, Bitincka L, Smalley J. MatGAT: An application that generates similarity/identity matrices using protein or DNA sequences. BMC Bioinformatics. 2003;4(29). doi: 10.1186/1471-2105-4-29 12854978 PMC166169

[22] Prakash A, Jeffryes M, Bateman A, Finn RD. The HMMER Web Server for Protein Sequence Similarity Search. Current Protocols in Bioinformatics. 2017;60(1):3.15.1–3.15.23. doi: 10.1002/cpbi.40 29220076

[23] Hu G, Kurgan L. Sequence Similarity Searching. Current Protocols in Protein Science. 2019;95(1):e71. doi: 10.1002/cpps.71 30102464

[24] Moret MA, Miranda JGV, Nogueira E, Santana MC, Zebende GF. Self-similarity and protein chains. Physical Review E, Statistical, Nonlinear, and Soft Matter Physics. 2005;71:012901. doi: 10.1103/PhysRevE.71.012901 15697638

[25] Souza LF, Rocha Filho TM, Moret MA. Relating SARS-CoV-2 variants using cellular automata imaging. Scientific Reports. 2022;12(10297). doi: 10.1038/s41598-022-14404-6 35717436 PMC9206224

[26] Souza JWG, Pereira HBB, Santos AAB, Senna V, Moret MA. A new proposal for analyzing combustion process stability based on the Hamming distance. Physica A. 2014;413:301–306. doi: 10.1016/j.physa.2014.06.057

[27] Tamura K, Stecher G, Kumar S. MEGA11: Molecular Evolutionary Genetics Analysis Version 11. Molecular Biology and Evolution. 2021;38(7):3022–3027. doi: 10.1093/molbev/msab120 33892491 PMC8233496

[28] Diao Y, Ma D, Wen Z, Yin J, Xiang J, Li M. Using pseudo amino acid composition to predict transmembrane regions in protein: cellular automata and Lempel-Ziv complexity. Amino Acids. 2008;34(1):111–117. doi: 10.1007/s00726-007-0550-z 17520325

[29] Pereira HBB, Zebende GF, Moret MA. Learning computer programming: Implementing a fractal in a Turing Machine. Computers & Education. 2010;55(2):767–776. doi: 10.1016/j.compedu.2010.03.009

[30] Xiao X, Chou KC. Digital Coding of Amino acids based on hydrophobic index. Protein and Peptide Letters. 2007;14(9):871–875. doi: 10.2174/092986607782110293 18045228

[31] Hamming RW. Error detecting and error correcting codes. The Bell System Technical Journal. 1950;29:147–160. doi: 10.1002/j.1538-7305.1950.tb00463.x

[32] Saw AK, Tripathy BC, Nandi S. Alignment-free similarity analysis for protein sequences based on fuzzy integral. Scientific Reports. 2019;9. doi: 10.1038/s41598-019-39477-8 30808983 PMC6391537

[33] Thompson JD, Higgins DG, Gibson TJ. CLUSTAL W: improving the sensitivity of progressive multiple sequence alignment through sequence weighting, position-specific gap penalties and weight matrix choice. Nucleic acids research. 1994;22(22):4673–4680. doi: 10.1093/nar/22.22.4673 7984417 PMC308517

[34] Cardol P. Mitochondrial NADH:ubiquinone oxidoreductase (complex I) in eukaryotes: A highly conserved subunit composition highlighted by mining of protein databases. Biochimica et Biophysica Acta (BBA)—Bioenergetics. 2011;1807(11):1390–1397. doi: 10.1016/j.bbabio.2011.06.015 21749854

[35] Saraçli S, Doğan N, Doğan İ. Comparison of hierarchical cluster analysis methods by cophenetic correlation. Journal of Inequalities and Applications. 2013;203:1–8.

[36] R Core Team. R: A Language and Environment for Statistical Computing; 2018. Available from: https://www.R-project.org/.

[37] Sokal RR, Rohlf FJ. The Comparison of Dendrograms by Objective Methods. Taxon. 1962;11(2):33–40. doi: 10.2307/1217208

[38] NCBI gene. MT-ND6 mitochondrially encoded NADH dehydrogenase 6 [homo sapiens (human)]—gene—NCBI; 2022. Available from: https://www.ncbi.nlm.nih.gov/gene/4541.

[39] Lambert LA, Perri H, Meehan TJ. Evolution of duplications in the transferrin family of proteins. Comparative Biochemistry and Physiology Part B: Biochemistry and Molecular Biology. 2005;140(1):11–25. doi: 10.1016/j.cbpc.2004.09.012 15621505

[40] Ford MJ. Molecular Evolution of Transferrin: Evidence for Positive Selection in Salmonids. Molecular Biology and Evolution. 2001;18(4):639–647. doi: 10.1093/oxfordjournals.molbev.a003844 11264416

[41] Aslan D, Crain K, Beutler E. A New Case of Human Atransferrinemia with a Previously Undescribed Mutation in the Transferrin Gene. Acta Haematologica. 2007;118:244–247. doi: 10.1159/000112726 18097132

[42] Hsia CCW. Respiratory Function of Hemoglobin. New England Journal of Medicine. 1998;338(4):239–248. doi: 10.1056/NEJM199801223380407 9435331

[43] Moret MA, Zebende GF. Amino acid hydrophobicity and accessible surface area. Phys Rev E. 2007;75:011920. doi: 10.1103/PhysRevE.75.011920 17358197

