# Supplementary material for: New distance measure for comparing protein using cellular automata image
Source: PLoS One. 2023 Oct 5;18(10):e0287880. doi: 10.1371/journal.pone.0287880 (PMC10553295; doi:10.1371/journal.pone.0287880)
Supplement: S1 File — (PDF) [file pone.0287880.s001.pdf]

Supporting information

All sequence data were taken from the NCBI genome database https://www.ncbi.nlm.nih.gov/protein. Below are the identifications of each species and protein used. All codes are available on GitHub: https://github.com/LuryaneF/protein-Similarities.

Table S1 . Identification of ND5 protein sequences at NCBI

Sequence name	Species	ID (NCBI)
Human	Homo sapiens	AP 000649.1
Gorilla	Gorilla gorilla	NP 008222.1
Common chimpanzee	Pan troglodytes	NP 008196.1
Pigmy chimpanzee	Pan paniscus	NP 008209.1
Fin whale	Balenoptera physalus	NP 006899.1
Blue whale	Balenoptera musculus	NP 007066.1
Rat	Rattus norvegicus	AP 004902.1
Mouse	Mus musculus	NP 904338.1
Opossum	Didelphis virginiana	NP 007105.1

Table S2 . Identification of ND6 protein sequences at NCBI.

Sequence name	Species	ID (NCBI)
Human	Homo sapiens	YP 003024037.1
Gorilla	Gorilla gorilla	NP 008223.1
Common chimpanzee	Pan troglodytes	NP 008197.1
Harbor seal	Phoca vitulina	NP 006939.1
Gray seal	Halichoerus grypus	NP 007080.1
Rat	Rattus norvegicus	AP 004903.1
Mouse	Mus musculus	NP 904339.1
Wallaroo	Osphranter robustus	NP 007405.1

Table S3 . Identification of Transferrin protein sequences at NCBI.

Sequence name	Species	ID (NCBI)
Human TF	Homo sapiens	S95936.1
Rabbit TF	Oryctolagus cuniculus	P19134.4
Rat TF	Rattus norvegicus	D38380.1
Cow TF	Bos Taurus	U02564.1
Buffalo LF	Bubalus bubalis	AJ005203.1
Cow LF	Bos Taurus	X57084.1
Goat LF	Capra hircus	X78902.1
Camel LF	Camelus dromedarius	AJ131674.1
Pig LF	Sus scrofa	AAA31102.1
Human LF	Homo sapiens	NM 002343.6
Mouse LF	Mus musculus	NM 008522.3
Possum TF	Trichosurus vulpecula	AF092510.1
Frog TF	Xenopus laevis	X54530.1
Japanese flounder TF	Paralichthys olivaceus	D88801.1
Atlantic salmon TF	Salmo salar	L20313.1
Brown trout TF	Salmo trutta	D89091.1
Lake trout TF	Salvelinus namaycush	D89090.1
Brook trout TF	Salvelinus fontinalis	D89089.1
Japanese char TF	Salvelinus leucomaenis pluvius	D89088.1
Chinook salmon TF	Oncorhynchus tshawytscha	AH008271.2
Coho salmon TF	Oncorhynchus kisutch	D89084.1
Sockeye salmon TF	Oncorhynchus nerka	D89085.1
Rainbow trout TF	Oncorhynchus mykiss	D89083.1
Amago salmon TF	Oncorhynchus masou	D89086.2

Table S4 . Identification of Beta-Globin protein sequences at NCBI.

Sequence name	Species	ID (NCBI)
Human	Homo sapiens	AAA16334.1
Pigeon	Columba Livia	P11342.1
Goshawk	Accipiter gentilis	P08851.1
Black Bear	Ursus thibetanus	P68012.1
Lesser Panda	Ailurus fulgens	P18982.1
Asiatic Elephant	Elephas maximus	P02084.1
Giant Panda	Ailuropoda melanoleuca	P18983.2
African Elephant	Loxodonta africana	P02085.1
Sheep	Ovis aries	P02075.2
Tortoise	Chelonoidis niger	P83123.3
Duck	Anas platyrhynchos	P02114.2
Grivet	Chlorocebus aethiops	P02028.1
Mallard	Anas platyrhynchos platyrhynchos	P02115.1
Gorilla	Gorilla gorilla gorilla	P02024.2
Goose	Anser anser anser	P02117.1
Shark	Heterodontus portusjacksoni	P02143.1
Rat	Rattus norvegicus	CAA33114.1
Hippopotamus	Hippopotamus amphibius	P19016.1
Penguin	Aptenodytes forsteri	P80216.1
Horse	Equus caballus	P02062.1
Swift	Apus apus	P15165.1
Gibbon	Hylobates lar	P02025.1
Coyote	Canis latrans	P60525.1
Whale	Balaenoptera acutorostrata	P18984.1
Catfish	Silurus asotus	O13163.2
Bat	Macroderma gigas	P24660.1
Bison	Bison bonasus	P09422.1
Red Fox	Vulpes vulpes	P21201.1
Swan	Cygnus olor	P68945.1
Marmot	Marmota marmota	P08853.1
Buffalo	Bubalus bubalis	P67820.1
Salmon	Salmo salar	Q91473.3
Dog	Canis lupus familiaris	P60524.1
Sparrow	Passer montanus	P07406.1
Chimpanzee	Pan troglodytes	P68873.2
Pheasant	Phasianus colchicus colchicus	P02113.1
Dolphin	Tursiops truncatus	P18990.1
Flamingo	Phoenicopterus ruber	P02121.1
Goldfish	Carassius auratus	P02140.1
Pig	Sus scrofa	P02067.3
Polar bear	Ursus maritimus	P68011.1
Dragonfish	Cygnodraco mawsoni	ADD73488.1
Rhinoceros	Rhinoceros unicornis	P09907.1
Parakeet	Psittacula krameri	P21668.1
Chicken	Gallus gallus	P02112.2
Zebra	Equus zebra	P67824.1
Wolf	Chrysocyon brachyurus	P60526.1
Cod	Gadus morhua	O13077.2
Turtle	Chrysemys picta bellii	P13274.1
Langur	Semnopithecus entellus	P02032.1
